# Detection of *Anaplasma phagocytophilum* and *Babesia aktasi* in a wild bezoar goat (*Capra aegagrus*): Overlap with domestic goat strains

**DOI:** 10.1111/mve.70003

**Published:** 2025-08-06

**Authors:** Aykut Zerek, Tuğba Özdemir, Maide Nur Gündoğdu, İpek Erdem, Ömer Orkun

**Affiliations:** ^1^ Department of Parasitology, Faculty of Veterinary Medicine Hatay Mustafa Kemal University Hatay Turkey; ^2^ Ticks and Tick‐Borne Diseases Research Laboratory, Department of Parasitology, Faculty of Veterinary Medicine Ankara University Ankara Turkey; ^3^ Graduate School of Health Sciences, Ankara University Ankara Turkey

**Keywords:** *Anaplasma phagocytophilum*, Anatolia, *Babesia aktasi*, piroplasms, tick‐borne pathogens, Turkey, Türkiye, wild goat

## Abstract

This study reports the first detection of *Babesia aktasi* and *Anaplasma phagocytophilum* in a bezoar goat (*Capra aegagrus*), providing insight into the presence of these pathogens in wild caprinae. The infected goat exhibited a localised ocular infection but showed no clinical signs of acute piroplasmid or *Anaplasma* infections. Microscopic examination of blood smears revealed low parasitemia of intra‐ and extraerythrocytic piroplasms and intragranulocytic morulae, consistent with chronic infection. PCR and sequence analysis confirmed that the *Babesia* species detected was *B. aktasi*, a recently described piroplasmid previously reported in domestic goats. Phylogenetic analysis placed the *B. aktasi* haplotype within the *Babesia* sensu stricto clade, closely related to sequences from domestic goats in Türkiye and an uncharacterised *Babesia* sp. from a red deer. The *A. phagocytophilum* strain detected in this study belonged to ecotype 1, which includes human pathogenic strains. These findings raise the possibility that bezoar goats may contribute to the natural maintenance of *B. aktasi* and *A. phagocytophilum*, highlighting their potential involvement in the enzootic cycles of these pathogens alongside domestic caprinae. Given that bezoar goats are the ancestors of modern domestic goats and that their habitats overlap in Anatolia, further research is needed to better understand the transmission dynamics, vector associations and zoonotic potential of these pathogens.

## Graphical abstract

- This study provides the first molecular detection of tick‐borne pathogens in *Capra aegagrus*.
- Genetic analysis reveals similarities between *Babesia aktasi* and *Anaplasma phagocytophilum* strains in bezoar and domestic goats, indicating potential pathogen exchange.
- This study expands our knowledge of tick‐borne pathogens in wild goat populations, highlighting the presence of pathogens not previously detected in this species.

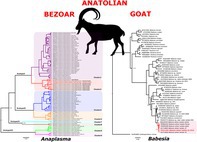

## INTRODUCTION

The bezoar goat (*Capra aegagrus* Erxleben, 1777) (Artiodactyla: Bovidae) (also known as the bezoar or bezoar ibex), the ancestor of today's domestic goat (*Capra hircus* Linnaeus, 1758), has existed and been distributed for thousands of years across a vast geography, ranging from the high mountains of southern and eastern Anatolia to Pakistan, along the Zagros Mountains of Iran (Weinberg & Ambarli, 2020; Zheng et al., 2020). The history of Anatolian people and the bezoar goat dates back to the Palaeolithic period. However, zooarchaeological and genetic data have revealed that Neolithic humans living in the Fertile Crescent, including Anatolia, began domesticating wild goats by adapting them to their environment around 10,500 years ago (Atici, 2009; Daly et al., 2018; Stiner et al., 2022). In addition to being an important ecological component, bezoar goats are closely related, both ecologically and genetically, to domestic goat herds in regions where both species are distributed (Pogorevc et al., 2024; Zheng et al., 2020). Therefore, the information gathered from wild goats in these regions has the potential to provide crucial insights into not only the domestication history of goats but also their pathogen/reservoir role and the pathogens they share with domestic goats. This could aid in better understanding both the evolutionary history and enzootic life cycles of the pathogens involved. On the other hand, while wild ungulates play a significant role as reservoirs in the enzootic life cycles of tick‐borne pathogens, such as *Anaplasma phagocytophilum* (Foggie, 1951) (Rickettsiales: Anaplasmataceace) (Jaarsma et al., 2019; Silaghi, Hamel, Thiel, et al., 2011; Stuen et al., 2013), identifying and, if necessary, controlling the pathogens that affect them is also important to ensure their conservation and population stability. Despite this, our understanding of the role of wild goats, particularly bezoar goats, in the enzootic life cycle of pathogens, especially tick‐borne pathogens, remains limited. For instance, morpho‐molecular characterisation of ticks (Acari: Ixodidae) collected from bezoar goats in Anatolia recently revealed the presence of certain tick species that had not been reported for a long time (mainly *Haemaphysalis kopetdaghica* Kerbabaev, 1962 and *Dermacentor raskemensis* Pomerantzev, 1946), and for the first time, these species were genetically barcoded (Orkun & Vatansever, 2021). However, the ecology of these ticks and the tick‐borne diseases associated with bezoar goats remain completely unknown. Therefore, data obtained from these wild goats are crucial for understanding their role in the maintenance and transmission of tick‐borne pathogens and for informing future wildlife health and conservation strategies.

The bezoar goat is a protected wild ungulate primarily inhabiting the mountains of regions of southern and eastern Anatolia. Considering the global population of the species, a substantial portion resides in this region (Weinberg & Ambarli, 2020; https://www.tramem.org/memeliler/?fsx=2fsdl17@d&tur=Yabankeçisi). Moreover, the natural habitats of these wild goats and the distribution areas of domestic goat herds overlap in many parts of Anatolia. Consequently, data obtained from bezoar goats in this area provide a valuable geographic context, not only to assess their status but also to investigate the pathogens they may share with domestic goats, including zoonotic ones. However, as sampling these animals is challenging, limited information exists regarding their role in the ecology of tick‐borne pathogens.

Several *Babesia* species (Piroplasmida: Babesiidae) have been identified in domestic goats worldwide, including *Babesia ovis* Babes, 1892, *Babesia motasi* Sergent & Sergent, 1912 and *Babesia crassa* Hashemi‐Fesharki and Uilenberg, 1981. These species differ in their geographic distribution, pathogenicity and host associations. While *B. ovis* is known to cause clinical disease in small ruminants—especially in sheep—other species are typically associated with subclinical or asymptomatic infections (Schnittger et al., 2022). By contrast, little is known about *Babesia* infections in wild caprines. The recently described *Babesia aktasi* Ozubek & Aktas, 2023 first identified in domestic goats in Türkiye (Ozubek, Ulucesme, & Aktas, 2023), has raised questions regarding its host range and epidemiological role. Providing context on the diversity of *Babesia* spp. in goats helps to clarify the rationale for investigating this pathogen in wild populations such as the bezoar goat. Therefore, this study aimed to molecularly identify and characterise tick‐borne pathogens detected in a bezoar goat captured in the southernmost part of Anatolia.

## MATERIALS AND METHODS

A young male bezoar goat (*C. aegagrus*), approximately 1.5 years old, living freely in the wild in the ‘İskenderun‐Arsuz Wildlife Development Area’ within the borders of Hatay Province, the southernmost province of Türkiye, was captured by national park officers on 20 December 2021. The animal was brought to Hatay Mustafa Kemal University Faculty of Veterinary Medicine for treatment. Initial examination revealed an ocular infection in both eyes of the animal, and eight engorged ticks were removed from the head and neck. The ticks collected were identified as *Ixodes gibbosus* Nuttall, 1916 (4♀ + 2♂), *Rhipicephalus kohlsi* Hoogstraal & Kaiser, 1960 (1♀) and *H. kopetdaghica* (1♀) based on morphological features, as described in detail in a previous publication (Zerek et al., 2023). A thorough clinical examination revealed no abnormalities other than the ocular infection. Since tick infestation was present, 3 mL of blood was collected from the jugular vein into a tube containing EDTA for routine diagnosis. Thin blood smears were prepared for microscopic examination, fixed in methanol for 5 min and stained with a 5% Giemsa solution (Giemsa's azur eosin methylene blue solution, Merck KGaA, Darmstadt, Germany) for 40 min. After Giemsa staining, the prepared slides were examined for the presence of protozoan and rickettsial pathogens using a light microscope with a 100× oil immersion objective. A Nikon Eclipse 80i (Nikon, Tokyo, Japan) light microscope equipped with a Leica MC170 HD digital camera (Leica, Heerbrugg, Switzerland) and LAS software (V4.11.0, Leica) was used for the microscopic examination. The blood sample was then sent to the Ticks and Tick‐Borne Diseases Research Laboratory (TTBDRL) at Ankara University, Faculty of Veterinary Medicine, for further analysis under appropriate conditions.

Genomic DNA was extracted from the EDTA‐anticoagulated whole blood collected from the bezoar goat using the PureLink™ Genomic DNA Mini Kit (Invitrogen, CA, USA) according to the manufacturer's protocol. The obtained gDNA was stored at −40°C until PCR analysis. The gDNA sample was tested using various conventional and nested PCR assays to detect the following tick‐borne pathogens: *Anaplasma* spp. (*Anaplasma marginale* Theiler, 1919*/Anaplasma ovis* Lestoquard, 1924 *A. phagocytophilum*) (de la Fuente et al., 2007), piroplasmids (Casati et al., 2006; Heidarpour Bami et al., 2009; Panait et al., 2021), *Borrelia burgdorferi* sensu lato (Sen et al., 2011), *Ehrlichia* spp. (Duscher et al., 2014), *Hepatozoon* spp. (Inokuma et al., 2002), *Coxiella burnetii* (Derrick, 1939) Philip, 1948 (Duron, 2015), *Neoehrlichia mikurensis* Kawahara et al., 2004 (Ondruš et al., 2020) and spotted fever group rickettsiae (Fournier et al., 1998; Roux et al., 1997). For the ecotype characterisation of *A. phagocytophilum*, an additional semi‐nested PCR assay amplifying a partial fragment of the heat shock protein gene (*groEL*) was performed (Alberti et al., 2005). All PCR primers, parameters and protocols used in this study are detailed in the Table S1. Each PCR run included a negative control (sterile DNase/RNase‐free water) and pathogen‐specific positive control DNA for various agents, including *A. phagocytophilum*, *A. ovis*, *Babesia divergens* M'Fadyean & Stockman, 1911, *Borrelia afzelii* Canica et al., 1994, *C. burnetii*, *Ehrlichia* sp., *Hepatozoon felis* Patton, 1908, *Rickettsia montanensis* corrig. (ex Lackman et al., 1965) Weiss & Moulder, 1984 and *Theileria annulata*. To optimise PCR conditions and avoid false negatives resulting from low target gene copy numbers, pre‐PCRs were conducted using positive controls at various dilutions (1:1–1:100 for conventional PCRs and 1:1–1:1000 for nested PCRs).

Amplified PCR products were purified using the ExoSAP‐IT™ PCR Product Cleanup Kit (Thermo Scientific, CA, USA). Sequencing was performed using the Sanger method with the BigDye™ Terminator v3.1 Cycle Sequencing Kit (Applied Biosystems, CA, USA) on an Applied Biosystems™ 3500 Genetic Analyzer. Raw sequence data obtained from both directions were examined, edited in the chromatograms and assembled into single consensus sequences using AliView v1.26 (Larsson, 2014). The assembled sequences were subjected to nucleotide similarity analysis (BLAST homology) against the GenBank database (NCBI, https://www.ncbi.nlm.nih.gov/genbank) for species identification. For phylogenetic analyses, datasets were constructed by combining the sequences determined in this study with GenBank records for related pathogens. In total, three datasets were generated: the 18S rRNA gene for piroplasmids (*Babesia* and *Theileria* spp.) and the *msp4* and *groEL* genes for *A. phagocytophilum*. Sequence reliability for each dataset was assessed using GUIDANCE2 (Sela et al., 2015), and unreliable sequences and columns (for protein‐coding genes) were excluded based on GUIDANCE2 outputs to improve the quality of phylogenetic analyses. The non‐coding gene (18S rRNA) was aligned using the Q‐INS‐i algorithm in MAFFT v7.475 (Katoh et al., 2019), which incorporates secondary structure information with a scoring matrix of 200 PAM/k = 2 and a gap opening penalty of 1.53. Protein‐coding genes (*msp4* and *groEL*) were aligned as translated amino acids using the MUSCLE algorithm (Edgar, 2004) integrated into AliView software. The overall mean distance (p‐distance) was calculated using MEGA v11.0.13 (Tamura et al., 2021) to estimate alignment confidence and sequence identity. Bayesian inference was performed for phylogenetic analyses using the Markov Chain Monte Carlo (MCMC) method in both MrBayes v3.2.6 (Ronquist et al., 2012) and the BEAST2 package (BEAUti v2.7.6, BEAST v2.7.6, TreeAnnotator v2.7.4) (Bouckaert et al., 2019). The best‐fitting nucleotide substitution models were selected based on the Bayesian information criterion using jModelTest 2.1.10 (Darriba et al., 2012). The MCMC chains were run for 10 million generations for MrBayes and 100 million generations for BEAST2, and ESS values were assessed using Tracer v1.7.2 (Rambaut et al., 2018). The maximum clade credibility (MCC) tree was generated using TreeAnnotator v2.7.4 after discarding the first 20% of trees as burn‐in. The final phylogenetic trees were visualised using FigTree v1.4.4 (Rambaut, A.; http://tree.bio.ed.ac.uk/software/figtree). Ecotypes and clusters of *A. phagocytophilum* were determined according to the classification proposed by Jaarsma et al. (2019) and Jahfari et al. (2014) based on *groEL* gene sequencing. Additionally, the nucleotide sequences of the pathogens obtained in this study were deposited in GenBank under specific accession numbers (PV211451 for *B. aktasi* and PV231891‐92 for *A. phagocytophilum*).

## RESULTS AND DISCUSSION

The clinical examination of the bezoar goat revealed a localised ocular infection in both eyes, and no other clinical signs or evidence of acute infection (in terms of piroplasmids and *Anaplasma* spp.) was found in the microscopic analysis of the blood films. Consequently, only the ocular infection was treated, following the routine treatment protocol, and the animal was subsequently released back into the wild after recovery.

Detailed PCR analysis of the blood sample for tick‐borne pathogens revealed positive amplicons of the expected sizes for three 18S rRNA PCRs targeting piroplasmids and the *msp4* gene of *A. phagocytophilum*. Additionally, the *groEL* gene of *A. phagocytophilum* was successfully amplified for ecotype analysis. Sequence analysis revealed that the positive amplicons belonged to *B. aktasi* and *A. phagocytophilum*.

**FIGURE 1:**
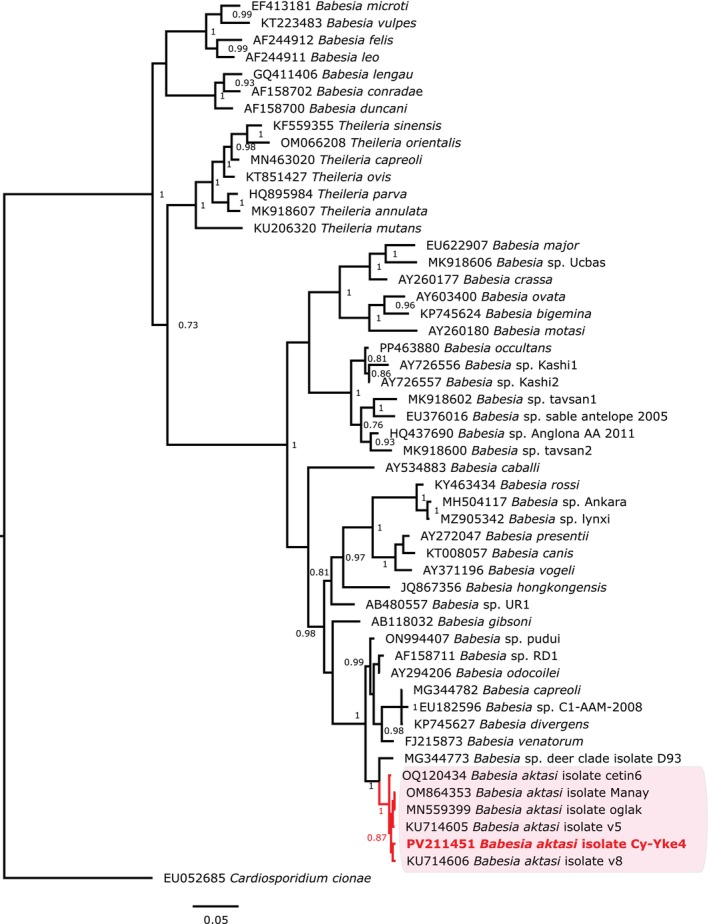
Phylogenetic tree constructed using Bayesian inference based on aligned nucleotide sequences of the 18S rRNA gene of piroplasms, with *Cardiosporidium cionae* Van Gaver & Stephan, 1907 (EU052685) as the outgroup, under the HKY+Γ+I substitution model. The analysis included 52 sequences and 1459 positions. Node labels indicate posterior probabilities, with values below 0.75 omitted. The haplotype sequence obtained in this study and its clade are highlighted in red. GenBank® accession numbers are provided before species names. The scale bar represents nucleotide substitutions per site.

BLAST homology analysis of the longest 18S rRNA gene sequence showed no identical sequences in GenBank, with 99.59% (1221/1226 bp) similarity to *B. aktasi* sequences obtained from domestic goats in Türkiye (MN559399 and OM864353). Furthermore, the sequence obtained from the bezoar goat also showed high similarity (98.04%, 1202/1226 bp) to an uncharacterised *Babesia* species (*Babesia* sp. deer, MG344773) detected in a red deer (*Cervus elaphus* Linnaeus, 1758) in the Czech Republic, representing the next closest match after *B. aktasi*. Bayesian inference phylogenetic analysis indicated that the *B. aktasi* haplotype detected in this study clustered within the main clade of *Babesia* sensu stricto and with other *B. aktasi* sequences obtained from domestic goats in Türkiye. Moreover, the *B. aktasi* clade was found to be monophyletic with the aforementioned ‘*Babesia* sp. deer’, with maximum posterior probability support (Figure 1). While initial microscopic examination did not reveal evidence of acute piroplasmid infection, a subsequent detailed re‐examination of the blood slides identified intra‐ and extraerythrocytic piroplasmic forms at very low parasitemia (<0.1%), suggesting chronic infection.

*Babesia aktasi* is a recently described species in domestic goats from the Mediterranean region of Türkiye, and the current knowledge of its biology and epidemiology remains limited (Ozubek, Ulucesme, & Aktas, 2023). Preliminary studies have reported a high prevalence of *B. aktasi*, particularly in southern Anatolia (Ulucesme et al., 2023), with low pathogenicity in indigenous domestic goats (Ozubek, Ulucesme, Bastos, et al., 2023). By contrast, experimental infections have shown that *B. aktasi* can be highly pathogenic in Saanen goats (Ulucesme et al., 2024a). The current study provides the first evidence of *B. aktasi* infection in a bezoar goat, suggesting that both wild and domestic goats may serve as susceptible hosts. However, the dynamics of pathogen maintenance and transmission between these populations remain unclear. Microscopic examination of blood smears revealed very low parasitemia levels in the absence of clinical signs. Considering the absence of acute clinical findings both at the time of capture and during the goat's temporary rehabilitation, these observations are consistent with a chronic infection. Notably, similar low parasitemia has been observed in experimentally infected domestic goats (Ozubek, Ulucesme, Bastos, et al., 2023). To determine whether wild bezoar goats act as true reservoirs or are incidental hosts of *B. aktasi*, further research is needed, including longitudinal surveillance and tick–host–pathogen interaction studies in wild populations.

Our understanding of *Babesia* species infecting wild goats, particularly bezoar goats, is still limited (Marco et al., 2000; Michel et al., 2014; Silaghi, Hamel, Pfister, & Rehbein, 2011). This study provides the first evidence of natural *Babesia* infection in a bezoar goat. Given that domestic goats are descendants of bezoar goats (Zheng et al., 2020) and that their habitats overlap in southern Anatolia, our findings suggest that both wild and domestic goats may serve as susceptible hosts for *B. aktasi*. However, the nature of potential pathogen circulation between these populations remains unclear and requires further investigation. Further studies on the diversity and host associations of *Babesia* spp., especially *B. aktasi*, in both wild and domestic caprines could improve our understanding of pathogen ecology and host–parasite coevolution. The competent vector for *B. aktasi* remains unknown, as the only vector competence study performed for this parasite demonstrated that *Rhipicephalus bursa* Canestrini & Fanzago, 1878 is not a competent vector (Ulucesme et al., 2024b). Given that the bezoar goat in this study was infested with *I. gibbosus*, *R. kohlsi* and *H. kopetdaghica*, future studies should first investigate whether these tick species are naturally infected with *B. aktasi*, before considering their potential role as vectors. It may be particularly useful to focus on *I. gibbosus*, a member of the *Ixodes ricinus* complex, given that *B. aktasi* is closely related to *B. divergens* and *Babesia venatorum*, both transmitted by species in the *I. ricinus* complex (primarily *I. ricinus* sensu stricto) (Rizzoli et al., 2014).

**FIGURE 2:**
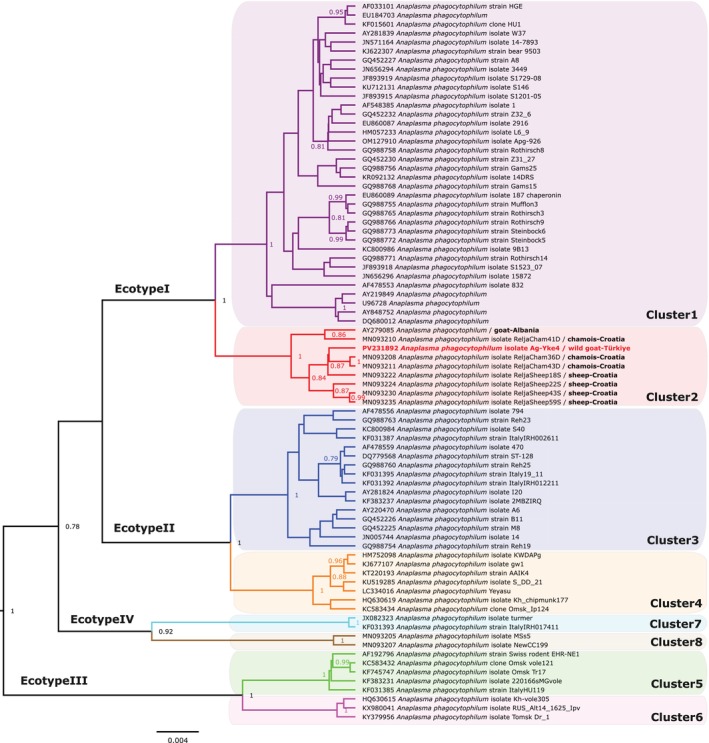
Phylogenetic tree constructed using Bayesian inference based on aligned nucleotide sequences of the *groEL* gene of *Anaplasma phagocytophilum* under the HKY+Γ+I substitution model. The analysis included 80 sequences and 530 positions. Node labels indicate posterior probabilities, with values below 0.75 omitted. The haplotype sequence obtained in this study and its clade are highlighted in red. GenBank® accession numbers are provided before species names. Collection source information with the origin country for each *A. phagocytophilum* sequence located in the ecotype1/cluster2 subclade is also given after isolate names. The scale bar represents nucleotide substitutions per site.

Both the *msp4* and *groEL* genes of *A. phagocytophilum*, another pathogen detected in the bezoar goat, were successfully amplified and sequenced. BLAST analysis of the *msp4* gene revealed 99.76% (822/824 bp) similarity with an *A. phagocytophilum* sequence (KY283959) obtained from a domestic goat in western Türkiye. Bayesian phylogenetic analysis of the *msp4* gene showed that the haplotype clustered within the main clade of *A. phagocytophilum*, together with sequences from a domestic goat in Türkiye (KY283959), a roe deer in Spain (AY829457) and an *I. ricinus* from Slovakia (HQ661160), with strong posterior probability support (Figure S1). Similarly, the *groEL* gene sequence exhibited 98.91% (542/548 bp) similarity with a sequence obtained from a domestic goat in Albania (AY279085). Phylogenetic analysis placed the haplotype within cluster 2 of ecotype 1, along with sequences from a domestic goat in Albania and from chamois and domestic sheep in Croatia (Figure 2). Microscopic examination of blood films from the bezoar goat revealed a low number of intragranulocytic morulae (<1% bacteremia), consistent with chronic infection.

This study represents the first report of *A. phagocytophilum* infection in a bezoar goat. *Anaplasma phagocytophilum* is a zoonotic bacterium with a broad host range and global distribution, primarily transmitted by *Ixodes* spp. Based on *groEL* gene diversity, four ecotypes and eight clusters have been described, with ecotype 1 comprising most strains associated with human infection (Jaarsma et al., 2019; Jahfari et al., 2014). Although the strain identified in this study belongs to cluster 2 of ecotype 1—rather than the more commonly zoonotic cluster 1—it is genetically close to cluster 1, which warrants further investigation into its zoonotic potential. While *A. phagocytophilum* has previously been documented in various wild ungulates (Jaarsma et al., 2019; Messner et al., 2019; Silaghi, Hamel, Thiel, et al., 2011; Stuen et al., 2013), this is the first evidence of infection in a bezoar goat. Our findings suggest that both wild and domestic goats may contribute to the ecology of ecotype 1/cluster 2 strains. However, additional data—particularly from wild populations—are needed to better understand the epidemiological dynamics and potential public health implications of this lineage. In Anatolia, although data on *A. phagocytophilum* are limited, different genotypes of the agent have been reported in domestic small ruminants and *I. ricinus* (Aktaş et al., 2021; Bilgic et al., 2017; Orkun, 2022). The current study adds to this knowledge by providing the first evidence of infection in a wild caprine host in Anatolia and contributes to our understanding of potential epidemiological links between wild and domestic goats.

In conclusion, this study is the first to report the presence of *A. phagocytophilum* and *B. aktasi* in bezoar goats, indicating that these pathogens are present in both wild and domestic goat populations in Anatolia. The close genetic relationship between the strains detected in this study and those previously reported from domestic goats underscores the need to investigate potential ecological links between wild and domestic caprinae. Further research is warranted to identify competent vectors of *B. aktasi*, clarify the reservoir status of wild goats and assess the zoonotic potential of *A. phagocytophilum* ecotype 1/cluster 2 strains.

## AUTHOR CONTRIBUTIONS

**Aykut Zerek:** Conceptualization; investigation; writing – review and editing. **Tuğba Özdemir:** Formal analysis; methodology; writing – review and editing. **Maide Nur Gündoğdu:** Methodology; writing – review and editing; formal analysis. **İpek Erdem:** Investigation; writing – review and editing. **Ömer Orkun:** Conceptualization; investigation; methodology; data curation; formal analysis; supervision; writing – original draft.

## CONFLICT OF INTEREST STATEMENT

The authors declare no conflicts of interest.

## ETHICS STATEMENT

The authors declare that all applicable international, national and/or institutional guidelines for care and use of wild animals were followed. This study was approved by the decision of the Hatay Mustafa Kemal University Animal Experiments Local Ethics Committee (2022/07‐08) and Directorate of Nature Conservation and National Parks (13 July 2023‐287402).

## Supporting information


**Figure S1.** Phylogenetic tree constructed using Bayesian inference based on aligned nucleotide sequences of the *msp4* gene of *Anaplasma phagocytophilum*, with *Anaplasma platys* (CP046391) as the outgroup, under the GTR+Γ+I substitution model. The analysis included 35 sequences and 813 positions. Node labels indicate posterior probabilities, with values below 0.75 omitted. The haplotype sequence obtained and its clade in this study are highlighted in red. GenBank® accession numbers are provided before species names. Collection source information with the origin country for each *A. phagocytophilum* sequence located in the clade is also given after isolate names. The scale bar represents nucleotide substitutions per site.

**Table S1.** PCR parameters for DNA amplification of target organisms.

## Data Availability

Sequencing data produced in the current study are available in GenBank (NCBI, https://www.ncbi.nlm.nih.gov/nucleotide/) under accession numbers PV211451 (*B. aktasi*) and PV231891‐92 (*A. phagocytophilum*).
