# Supplementary figures and images for: Detection of Anaplasma phagocytophilum and Babesia aktasi in a wild bezoar goat (Capra aegagrus): Overlap with domestic goat strains
Source: Med Vet Entomol. 2025 Aug 6;40(1):190–7. doi: 10.1111/mve.70003 (PMC12865739; doi:10.1111/mve.70003)

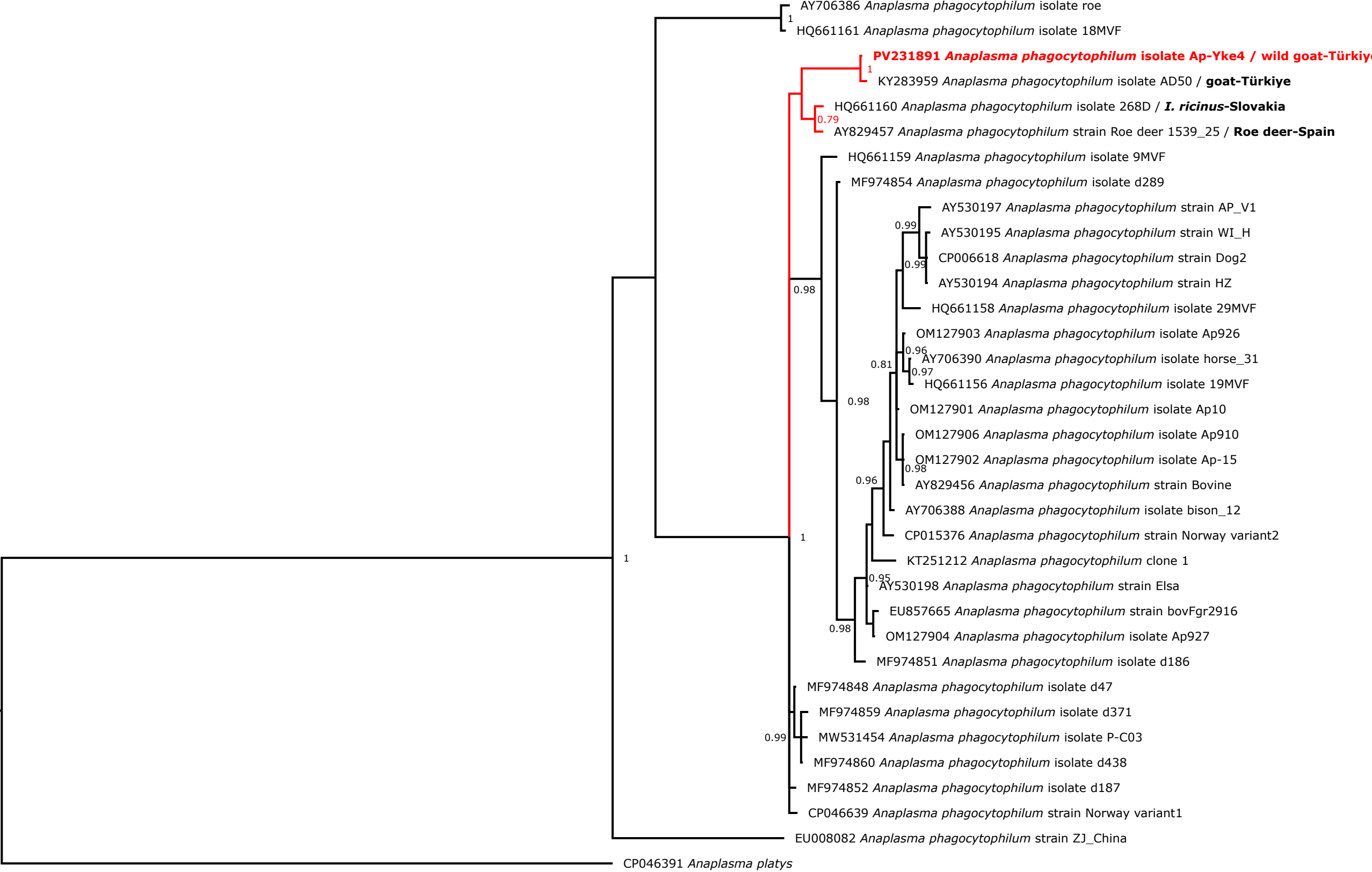

0.05

Supplement: Supplementary file 1 — Figure S1. Phylogenetic tree constructed using Bayesian inference based on aligned nucleotide sequences of the msp4 gene of Anaplasma phagocytophilum, with Anaplasma platys (CP046391) as the outgroup, under the GTR+Γ+I substitution model. The analysis included 35 sequences and 813 positions. Node labels indicate posterior probabilities, with values below 0.75 omitted. The haplotype sequence obtained and its clade in this study are highlighted in red. GenBank® accession numbers are provided before species names. Collection source information with the origin country for each A. phagocytophilum sequence located in the clade is also given after isolate names. The scale bar represents nucleotide substitutions per site. [file MVE-40-190-s001.pdf]
