# Supplementary material for: Detection of Anaplasma phagocytophilum and Babesia aktasi in a wild bezoar goat (Capra aegagrus): Overlap with domestic goat strains
Source: Med Vet Entomol. 2025 Aug 6;40(1):190–7. doi: 10.1111/mve.70003 (PMC12865739; doi:10.1111/mve.70003)
Supplement: Supplementary file 2 — Table S1. PCR parameters for DNA amplification of target organisms. [file MVE-40-190-s002.docx]

**Supplementary Table.** PCR parameters for DNA amplification of target organisms

| Target organism | Target gene  region | Type of PCR | Primers (F, R) | Product  size (bp) | Cycling parameters | Reference |
| --- | --- | --- | --- | --- | --- | --- |
| Piroplasms | 18S rRNA | Conventional | BJ1  BN2 | 411-452 | 94 °C 2 min, 40 cycles [94 °C 30 s, 55 °C 30 s, 72 °C 1 min], 72°C 10 min | Casati et al.,  2006 |
|  | 18S rRNA | Nested | Thei F1 – Thei R1  Thei F2 – Thei R2 | ~1700  1417-1426 | 94 °C 2 min, 35 cycles [94 °C 30 s, 50 °C 30 s, 72 °C 2 min], 72°C 10 min (the first round)  94 °C 2 min, 35 cycles [94 °C 30 s, 52 °C 30 s, 72 °C 1 min:30 s] 72°C 10 min (the second round) | Heidarpour Bami et al., 2009 |
|  | 18S rRNA | Nested | 7549F–  7548R  Cyt-SSU-F2–  Cyt-SSU-R4 | 1726  1335 | 94 °C 2 m, 35 cycles [94 °C 30 s, 60 °C 30 s, 72 °C 2 min], 72°C 10 min (the first round)  94 °C 2 m, 35 cycles [94 °C 30 s, 53 °C 30 s, 72 °C 1 min:30 s] 72°C 10 min (the second round) | Panait et al., 2021 |
| *Hepatozoon* spp. | 18S rRNA | Conventional | HepF  HepR | ~666 | 94 °C 2 min, 40 cycles [94 °C 30 s, 55 °C 30 s, 72 °C 1 min], 72°C 10 min | Inokuma et al., 2002 |
| *Anaplasma*  *marginale*/*A. ovis* | *msp4* | Conventional | MSP45  MSP43 | 851 | 94 °C 2 min, 40 cycles [94 °C 30 s, 60 °C 30 s, 72 °C 1 min], 72°C 10 min | de la Fuente et  al., 2007 |
| *Anaplasma phagocytophilum* and its variants | *msp4* | Conventional | MAP4AP5  MSP4AP3 | 849 | 94 °C 2 min, 40 cycles [94 °C 30 s, 54 °C 30 s, 72 °C 1 min], 72°C 10 min | de la Fuente et  al., 2007 |
|  | *groEL* | Semi-nested | EphplgroEL(569)F–  EphplgroEL(1193)R  EphplgroEL(569)F–  EphgroEL(1142)R | 624  573 | 94 °C 2 min, 35 cycles [94 °C 30 s, 54 °C 30 s, 72 °C 1 min], 72°C 10 min (the first round)  94 °C 2 min, 35 cycles [94 °C 30 s, 54 °C 30 s, 72 °C 1 min 72°C 10 min (the second round) | Alberti et  al., 2005 |
| *Rickettsia* spp. | *gltA* | Conventional | *Rp* CS.409d  *Rp* CS. 1258n | 750 | 94 °C 2 min, 40 cycles [94 °C 30 s, 54 °C 30 s, 72 °C 1 min], 72°C 10 min | Roux et al.,  1997 |
|  | *ompA* | Conventional | Rr. 190.70  Rr. 190.701 | 629-632 | 94 °C 2 min, 40 cycles [94 °C 30 s, 53 °C 30 s, 72 °C 1 min], 72°C 10 min | Fournier et al.,  1998 |
| *Borrelia burgdorferi* sensu lato | 5S-23S rRNA | Nested | RIS1 – RIS2  RIS3 – RIS4 | 226-266  ~200 | 94 °C 2 min, 35 cycles [94 °C 30 s, 52 °C 30 s, 72 °C 1 min], 72°C 10 min (the first round)  94 °C 2 min, 35 cycles [94 °C 30 s, 50 °C 30 s, 72 °C 1 min] 72°C 10 min (the second round) | Şen et al., 2011 |
| *Ehrlichia* spp. | 16S rRNA | Conventional | Ehr.u.for  Ehr.CCE.rev | 619 | 94 °C 2 min, 40 cycles [94 °C 30 s, 61 °C 30 s, 72 °C 1 min], 72°C 10 min | Duscher et al., 2014 |
| *Neoehrlichia mikurensis* | *groEL* | Conventional | CNM_groEL_PCR_F  CNM_groEL_PCR_R | 654 | 94 °C 2 min, 40 cycles [94 °C 30 s, 52.4 °C 30 s, 72 °C 1 min], 72°C 10 min | Ondrus et al., 2020 |
| *Coxiella burnetii* | IS1111-Tnp | Nested | IS1111_F1-IS1111_R2  IS1111_F2-IS1111_R1 | 670  570 | 94 °C 2 min, 35 cycles [94 °C 30 s, 56 °C 30 s, 72 °C 1 min], 72°C 10 min (the first round)  94 °C 2 min, 35 cycles [94 °C 30 s, 56 °C 30 s, 72 °C 1 min] 72°C 10 min (the second round) | Duron, 2015 |
